# Use of GLP-1 Receptor Agonists and SGLT2 Inhibitors Among Patients with Type 1 Diabetes: A Nationwide Register-Based Cohort Study

**DOI:** 10.1016/j.lanepe.2026.101729

**Published:** 2026-06-04

**Authors:** Carl-Emil Lim, Björn Pasternak, Björn Eliasson, Laura Pazzagli, Peter Ueda

**Affiliations:** a Centre for Pharmacoepidemiology, Division of Clinicial Epidemiology, Department of Medicine, Solna, Karolinska Institutet, Stockholm, Sweden; b Department of Data Science and AI in Health, Statens Serum Institut, Copenhagen, Denmark; c Department of Medicine, Sahlgrenska University Hospital, Gothenburg, Sweden

**Keywords:** Type 1 diabetes, GLP-1 receptor agonists, SGLT2 inhibitors, Off-label use

## Abstract

**Background:**

GLP-1 receptor agonists and SGLT2 inhibitors improve cardiometabolic risk factors in type 1 diabetes, but lack approval, partly due to safety concerns. We aimed to assess the use of these drugs in a national population of patients with type 1 diabetes.

**Methods:**

Using Swedish nationwide registers, we included all patients aged ≥18 years with type 1 diabetes, 2007–2024. We calculated yearly prevalence of GLP-1 receptor agonist and SGLT2 inhibitor use based on prescription fills; the proportion of patients with elevated HbA1c or obesity, without receiving any of the drugs; and diabetic ketoacidosis incidence among SGLT2 inhibitor users.

**Findings:**

72,698 patients were included (median (IQR) age in 2024: 48 (33, 63) years). Use of the drugs increased during the study period and reached 5.5% (3220/58,564) in 2024 for GLP-1 receptor agonists and 0.9% (552/58,564) for SGLT2 inhibitors (peak in 2022: 1.1% [610/55,949]). In 2024, 24.5% (14,348/58,564) of patients had HbA1c of ≥64 mmol/mol (≥8.0%) and 16.8% (9839/58,564) had obesity and were not treated with either drug. Among 1291 first-time SGLT2 inhibitor users, the on-treatment incidence of diabetic ketoacidosis was 24.9 (95% CI 18.4–31.5) events per 1000 person-years.

**Interpretation:**

Use of GLP-1 receptor agonists and SGLT2 inhibitors in type 1 diabetes remained low (5.5% and 0.9% in 2024), with many untreated patients having suboptimal cardiometabolic risk factors. While ancillary treatment could potentially improve outcomes, regulatory approval is lacking and further research on the risk-benefit balance across clinically relevant subgroups would help inform potential expansion of use.

**Funding:**

The Swedish Research Council, Swedish Society of Medicine and ALF Region Stockholm.

**Research in context**

**Evidence before this study**

We searched for population-based studies assessing the proportion of patients with type 1 diabetes using SGLT2 inhibitors or GLP-1 receptor agonists. We searched PubMed for articles in all languages published through March 4, 2025, using the search terms “(“Diabetes Mellitus, Type 1”[Mesh] OR “Type 1 Diabetes”[tiab] OR “T1DM”[tiab] OR “insulin-dependent diabetes”[tiab]) AND (“Sodium-Glucose Transporter 2 Inhibitors”[Mesh] OR “SGLT2 inhibitor∗”[tiab] OR “SGLT-2 inhibitor∗:”[tiab] OR “gliflozin∗”[tiab] OR “Glucagon-Like Peptide-1 Receptor Agonists”[Mesh] OR “GLP-1 receptor agonist∗”[tiab] OR “GLP-1 agonist∗”[tiab] OR “GLP-1 RA”[tiab])”. We also assessed all articles in which identified articles had been cited. We found no population-based studies assessing the use of GLP-1 receptor agonists or SGLT2 inhibitors, or factors associated with their use in type 1 diabetes. One study from the US based on electronic health records (n = 405,019), reported that 6.6% of patients with type 1 diabetes were prescribed a GLP-1 receptor agonist and 2.4% were prescribed an SGLT2 inhibitor in 2023. European studies were limited by small or selected study populations.

**Added value of this study**

In this nationwide cohort of 72,698 adults with type 1 diabetes in Sweden, use of GLP-1 receptor agonists and SGLT2 inhibitors increased between 2007 and 2024 but remained low (5.5% and 0.9% in 2024). For both drug classes, use was associated with older age, higher BMI, higher HbA1c, chronic kidney disease and cardiovascular comorbidities, with stronger associations for the latter two for SGLT2 inhibitors. In 2024, 24.5% of patients had HbA1c ≥ 64 mmol/mol and 16.8% had obesity without treatment with either drug class. Among 1291 first-time users of an SGLT2 inhibitor, the on-treatment incidence rate of diabetic ketoacidosis was 24.9 [95% CI 18.4–31.5] per 1000 person-years.

**Implications of all the available evidence**

The evidence indicates that the use of GLP-1 receptor agonists and SGLT2 inhibitors in patients with type 1 diabetes have increased over recent years but remains low. It is possible that cardiometabolic risk factors among type 1 diabetes patients could be improved using ancillary treatment with SGLT2 inhibitors and GLP-1 receptor agonists. However, regulatory approval for this indication is currently lacking and the balance of risks and benefits across clinically relevant subgroups remains incompletely understood. In particular, strategies for safe use need to be investigated, especially with respect to the diabetic ketoacidosis risk associated with SGLT2 inhibitors.

## Introduction

Glucagon-like peptide-1 (GLP-1) receptor agonists and sodium-glucose cotransporter-2 (SGLT2) inhibitors are key treatments for type 2 diabetes but lack regulatory approval for type 1 diabetes. Clinical trials in patients with type 1 diabetes of both drug classes have shown benefits in improving glycaemic control, reducing insulin requirements and promoting weight loss.^1^, ^2^, ^3^, ^4^

When added to insulin, patients treated with liraglutide in the ADJUNCT ONE and ADJUNCT TWO trials^2^^,^^3^ or with semaglutide in the ADJUST-T1D trial,^1^ showed improved glycaemic control and weight loss compared to placebo. However, in the ADJUNCT ONE and ADJUNCT TWO trials, patients receiving liraglutide had an increased risk of hypoglycemia and hyperglycemia with ketosis, although similar risk increases were not observed in the smaller trial of semaglutide. Similarly, clinical trials of SGLT2 inhibitors (empagliflozin, dapagliflozin and sotagliflozin) as adjunct therapy to insulin in patients with type 1 diabetes, showed reduced HbA1c levels and body weight but substantially increased rates of diabetic ketoacidosis compared to placebo (0.8–6.0% vs. 0.0–1.2%), despite efforts to mitigate this risk.^4^

Due to safety concerns for diabetic ketoacidosis, no SGLT2 inhibitor is currently approved by the US Food and Drug Administration for type 1 diabetes.^5^ The European Medicines Agency (EMA) approved dapagliflozin and sotagliflozin for selected patients with type 1 diabetes (BMI ≥27 kg/m^2^, inadequate glycaemic control despite optimal insulin treatment, requiring increased surveillance of ketone levels) in 2019^6^^,^^7^ but later withdrew the approval for dapagliflozin in 2021^8^ and for sotagliflozin in 2022.^9^

While the role of SGLT2 inhibitors and GLP-1 receptor agonists in the treatment of type 1 diabetes remains debated,^4^^,^^10^, ^11^, ^12^, ^13^, ^14^ data from routine clinical practice show that both drugs are being used in patients with type 1 diabetes.^15^, ^16^, ^17^, ^18^ A study from the US,^16^ analyzing electronic health record data including 405,019 patients with type 1 diabetes in 2023, showed that 6.6% were prescribed a GLP-1 receptor agonist and 2.4% prescribed an SGLT2 inhibitor. Studies from Europe have been limited by small or selected study populations, and large population-based studies are lacking.^17^^,^^18^ Thus, population-based data on ancillary treatment with these drugs in type 1 diabetes are needed to quantify the extent of off-label prescribing in routine care, characterize patient selection and assess potentially unmet clinical needs.

We used nationwide registers in Sweden including all patients with type 1 diabetes to assess the use trends for GLP-1 receptor agonists and SGLT2 inhibitors, the association of patient characteristics with use of the drugs, the proportion of patients who had elevated HbA1c or obesity without receiving any of the drugs, and to describe the incidence of diabetic ketoacidosis among SGLT2 inhibitor users.

## Methods

### Study design and participants

We conducted an open cohort study including all patients aged 18 years or older with type 1 diabetes in Sweden between year 2007 and 2024. Each year in the study period was assessed separately. Patients were categorized as having type 1 diabetes if they had at least one type 1 diabetes diagnosis (ICD-10: E10) registered at a specialist care outpatient clinic visit in the National Patient Register in the 3 years prior the study year, had no type 2 diabetes diagnosis registered during or prior to the study year in either the National Patient Register (inpatient or outpatient) or the National Diabetes Register, and had at least one dispensation of insulin in the year prior the study year. Patients who died or emigrated prior to or during the study year were excluded.

### Data sources

Data on study drugs and co-medications was obtained from the Swedish National Prescribed Drug Register, which holds individual-level information on all drug prescriptions filled at all pharmacies in Sweden since July 2005. The register includes information on the anatomical therapeutic chemical code of the dispensed drug, item number, the amount of drug dispensed, dosage form (pill or injection), and the date when the prescription was filled.^19^

We obtained data on HbA1c level, blood pressure, estimated glomerular filtration rate (eGFR), albuminuria, body mass index and smoking status from the Swedish National Diabetes Register. For this nationwide register, data are collected by physicians and nurses during visits to outpatient and primary care clinics by patients with type 1 or type 2 diabetes in Sweden.^20^

We obtained data on procedure codes and diagnoses according to the International Classification of Diseases, tenth revision (ICD-10) from the National Patient Register. These are assigned by physicians during hospital admissions and outpatient specialist care visits in Sweden.^21^

Individual-level data on age, sex, place of birth, educational level and income were obtained from the Total Population Register, which is maintained by Statistics Sweden.^22^ Data on vital status were obtained from The Cause of Death register^23^ and data on educational level and income were obtained from the longitudinal integrated database for health insurance and labour market studies (LISA).^24^

Individual consent is not required for patients to be included in national health registries such as the National Diabetes register (but opt out is possible), or this study, according to Swedish law. The study was approved by the Swedish Ethical Review Authority on 13-10-2021 (registration number: 2021–03957).

### Statistical analyses

We calculated the yearly prevalence of GLP-1 receptor agonist and SGLT2 inhibitor use in the overall population across all study years (2007–2024). Use of a study drug was defined as having filled at least one prescription for either drug during the examined study year. We also calculated the yearly prevalence for individual drugs within each study drug class. ATC codes for all study drugs are provided in Supplementary Table S1.

For each study year, we assessed the prevalence in the total study population of those who did not receive GLP-1 receptor agonists or SGLT2 inhibitors by joint categories of obesity (BMI ≥30 kg/m^2^) status (yes/no) and HbA1c level (<53 mmol/mol; ≥53 mmol/mol (≥7.0%) to <64 mmol/mol (<8.0%); HbA1c ≥ 64 mmol/mol (≥8.0%)). These analyses were performed to assess the proportion of patients who potentially could benefit from ancillary treatment with GLP-1 receptor agonists or SGLT2 inhibitors. For the year 2024, we described the population characteristics by status of treatment with GLP-1 receptor agonists and SGLT2 inhibitors. Given the proportions of missing values for albuminuria (33.1%), BMI (30.2%), eGFR (29.7%), blood pressure (21.9%), smoking (22.1%), HbA1c (16.9%), living with partner (<1%), education (<1%), income (<1%), and place of birth (<1%), we used multiple imputation (10 imputed datasets) to handle missing data, assuming data were missing at random conditionally on the variables included in the imputation model.^25^^,^^26^ The imputation model included all variables in Table 1 and the outcome variables (use of GLP-1 receptor agonists and SGLT2 inhibitors). All analyses that included variables with imputed values were run across all imputed datasets and then pooled in accordance with Rubin’s rules.^27^

**Table 1:** Patient characteristics and odds ratios (OR) for use of GLP-1 receptor agonist and SGLT2 inhibitor in 2024.

Characteristic					
Sociodemographic characteristics	Overall, n = 58,564	GLP-1 receptor agonists, n = 3220	OR (95% CI)	SGLT2 inhibitors, n = 552	OR (95% CI)
Women	25,879 (44.2)	1819 (56.5)	1.73 (1.61–1.86)	233 (42.2)	0.88 (0.74–1.04)
Age					
<20	1779 (3.0)	26 (0.8)	Ref	3 (0.5)	Ref
20–29	9081 (15.5)	236 (7.3)	1.81 (1.23–2.79)	8 (1.4)	0.52 (0.15–2.38)
30–39	10,920 (18.6)	564 (17.5)	3.70 (2.54–5.64)	27 (4.9)	1.47 (0.52–6.14)
40–49	8840 (15.1)	660 (20.5)	5.53 (3.8–8.41)	65 (11.8)	4.37 (1.62–17.91)
50–59	9960 (17.0)	856 (26.6)	6.40 (4.41–9.73)	110 (19.9)	6.61 (2.49–26.85)
60–69	8741 (14.9)	644 (20.0)	5.37 (3.7–8.18)	142 (25.7)	9.78 (3.71–39.68)
≥70	9243 (15.8)	234 (7.3)	1.71 (1.16–2.64)	197 (35.7)	12.96 (4.93–52.47)
Place of birth					
Nordic countries	54,100 (92.4)	2911 (90.4)	Ref	495 (89.7)	Ref
Outside Europe	2698 (4.6)	194 (6.0)	1.29 (1.11–1.51)	40 (7.2)	2.11 (1.52–2.94)
Rest of Europe	1766 (3.0)	115 (3.6)	1.10 (0.91–1.34)	17 (3.1)	1.15 (0.71–1.87)
Education					
Primary school and high school	35,671 (60.9)	2014 (62.6)	Ref	407 (73.7)	Ref
Vocational or short-term tertiary education	9112 (15.6)	526 (16.3)	0.93 (0.84–1.03)	59 (10.7)	0.61 (0.45–0.82)
Medium or long tertiary education	13,781 (23.5)	680 (21.1)	0.69 (0.63–0.76)	86 (15.6)	0.59 (0.46–0.75)
Income					
Low income	15,016 (25.6)	833 (25.9)	Ref	173 (31.3)	Ref
Middle income	29,013 (49.5)	1579 (49.0)	0.98 (0.90–1.07)	259 (47.0)	0.77 (0.63–0.95)
High income	14,535 (24.8)	808 (25.1)	1.01 (0.91–1.12)	120 (21.7)	0.72 (0.56–0.91)
Household income					
Low income	14,743 (25.2)	778 (24.1)	Ref	161 (29.2)	Ref
Middle income	29,119 (49.7)	1642 (51.0)	1.07 (0.98–1.18)	270 (48.8)	0.84 (0.69–1.03)
High income	14,702 (25.1)	800 (24.9)	1.04 (0.93–1.15)	121 (22.0)	0.75 (0.58–0.96)
Living with partner	21,678 (37.0)	1428 (44.3)	1.14 (1.06–1.23)	280 (50.7)	1.08 (0.91–1.28)
Medical history (10-year look-back)					
Cardiovascular disease	6302 (10.8)	427 (13.3)	1.47 (1.31–1.64)	199 (36.1)	2.69 (2.22–3.25)
Ischemic heart disease and coronary revascularization	3769 (6.4)	278 (8.6)	1.64 (1.43–1.88)	151 (27.4)	3.10 (2.53–3.79)
Ischemic stroke	1241 (2.1)	77 (2.4)	1.33 (1.04–1.67)	33 (6.0)	1.59 (1.09–2.24)
Arterial disease (including amputation)	2745 (4.7)	161 (5.0)	1.15 (0.97–1.36)	74 (13.4)	1.83 (1.41–2.35)
Atrial fibrillation	1943 (3.3)	100 (3.1)	1.35 (1.08–1.66)	102 (18.5)	3.74 (2.94–4.73)
Heart failure	1609 (2.7)	93 (2.9)	1.32 (1.05–1.63)	117 (21.2)	5.81 (4.63–7.25)
Chronic kidney disease	5532 (9.4)	342 (10.6)	1.20 (1.06–1.37)	137 (24.9)	1.90 (1.52–2.37)
Liver disease	821 (1.4)	60 (1.9)	1.26 (0.95–1.63)	16 (2.9)	1.68 (0.97–2.69)
Pancreatitis	283 (0.5)	15 (0.5)	1.00 (0.57–1.63)	9 (1.6)	2.81 (1.33–5.2)
Ketoacidosis	6063 (10.4)	308 (9.6)	1.08 (0.95–1.22)	30 (5.4)	0.77 (0.52–1.1)
Diabetic eye complications	37,990 (64.9)	2330 (72.4)	1.17 (1.07–1.27)	384 (69.6)	0.77 (0.64–0.93)
Other diabetic complications	11,078 (18.9)	625 (19.4)	1.16 (1.06–1.27)	96 (17.4)	0.99 (0.79–1.23)
Psychiatric disorder	10,896 (18.6)	749 (23.3)	1.40 (1.28–1.53)	88 (15.9)	1.14 (0.9–1.43)
Mental and behavioral disorders due to psychoactive substance use	3399 (5.8)	193 (6.0)	1.07 (0.92–1.24)	33 (6.0)	1.21 (0.83–1.69)
Medications in the previous year					
Previous use of GLP-1 receptor agonist	2958 (5.1)	2372 (73.7)	–	126 (22.8)	5.82 (4.71–7.14)
Previous use of SGLT2 inhibitor	621 (1.1)	159 (4.9)	6.21 (5.12–7.50)	416 (75.4)	–
Metformin	2870 (4.9)	703 (21.8)	6.38 (5.79–7.03)	141 (25.5)	5.48 (4.48–6.66)
DPP4 inhibitors	243 (0.4)	29 (0.9)	2.29 (1.51–3.35)	22 (4.0)	7.42 (4.60–11.40)
Sulfonylureas	16 (0.0)	3 (0.1)	2.88 (0.66–9.06)	<3 (0.4)	14.69 (2.27–54.25)
Other non-insulin diabetes drugs (glitazones, glinides, acarbose)	33 (0.1)	7 (0.2)	4.04 (1.59–9.00)	3 (0.5)	7.65 (1.82–21.87)
Platelet inhibitors	1435 (2.5)	103 (3.2)	1.44 (1.17–1.77)	43 (7.8)	1.85 (1.33–2.52)
Statins	27,136 (46.3)	2015 (62.6)	2.14 (1.96–2.33)	442 (80.1)	2.15 (1.71–2.73)
ACE inhibitors/Angiotensin II receptor blockers	21,491 (36.7)	1649 (51.2)	2.16 (1.99–2.35)	401 (72.6)	2.33 (1.89–2.89)
Calcium antagonists	10,426 (17.8)	767 (23.8)	1.65 (1.50–1.81)	198 (35.9)	1.33 (1.10–1.60)
Beta-blockers	8831 (15.1)	749 (23.3)	2.02 (1.84–2.21)	274 (49.6)	3.46 (2.88–4.15)
Diuretics	5772 (9.9)	517 (16.1)	2.10 (1.88–2.33)	201 (36.4)	3.07 (2.54–3.71)
Time since first diabetes drug					
<3 year	1582 (2.7)	55 (1.7)		14 (2.5)	Ref
≥3 to <10 years	8858 (15.1)	442 (13.7)	1.16 (0.98–1.36)	70 (12.7)	0.92 (0.63–1.37)
≥10 years	48,124 (82.2)	2723 (84.6)	1.04 (0.90–1.20)	468 (84.8)	0.60 (0.44–0.85)
Number of diabetes drugs					
1 or 2	49,150 (83.9)	972 (30.2)	Ref	127 (23.0)	Ref
≥3	9414 (16.1)	2248 (69.8)	14.91 (13.76–16.16)	425 (77.0)	16.74 (13.74–20.54)
National Diabetes Register variables (previous year)					
Insulin pump use^a^	16,993 (29.0)	938 (29.1)	0.99 (0.91–1.07)	78 (14.1)	0.61 (0.47–0.78)
Blood pressure					
Normotension	45,286 (77.3)	2420 (75.2)	Ref	398 (72.2)	Ref
Stage 1 hypertension	11,422 (19.5)	693 (21.5)	1.11 (1.01–1.23)	125 (22.6)	0.87 (0.68–1.1)
Stage 2 hypertension	1856 (3.2)	107 (3.3)	1.14 (0.92–1.41)	28 (5.2)	1.05 (0.7–1.56)
HbA1c					
≤48 mmol/mol (≤6.5%)	13,720 (23.4)	532 (16.5)	Ref	80 (14.4)	Ref
49–52 mmol/mol (6.6–6.9%)	8462 (14.5)	402 (12.5)	1.20 (1.04–1.39)	74 (13.3)	1.35 (0.96–1.90)
53–63 mmol/mol (7.0–7.9%)	20,748 (35.4)	1161 (36.0)	1.42 (1.26–1.59)	198 (35.8)	1.38 (1.03–1.84)
64–74 mmol/mol (8.0–8.9%)	9972 (17.0)	713 (22.2)	1.87 (1.64–2.13)	129 (23.3)	1.77 (1.32–2.38)
≥75 mmol/mol (≥9.0%)	5661 (9.7)	412 (12.8)	1.96 (1.67–2.30)	73 (13.2)	1.99 (1.42–2.78)
Body mass index					
Normal weight: <25 kg/m^2^	24,255 (41.4)	239 (7.4)	Ref	117 (21.3)	Ref
Overweight: ≥25 to <30 kg/m^2^	22,244 (38.0)	908 (28.2)	4.07 (3.36–4.92)	209 (37.8)	1.69 (1.31–2.17)
Obese class I: ≥30 to <35 kg/m^2^	8761 (15.0)	1154 (35.8)	13.97 (11.26–17.34)	142 (25.7)	2.87 (2.2–3.73)
Obese class II/III: ≥35 kg/m^2^	3304 (5.6)	919 (28.5)	33.80 (25.63–44.58)	84 (15.2)	5.22 (3.71–7.33)
eGFR (ml/min per 1.73m^2^)					
≥90	31,768 (54.2)	1668 (51.8)	Ref	184 (33.4)	Ref
60 to <90	22,128 (37.8)	1281 (39.8)	0.91 (0.83–0.99)	249 (45.2)	1.13 (0.89–1.43)
30 to <60	3975 (6.8)	228 (7.1)	1.07 (0.91–1.27)	103 (18.6)	1.97 (1.51–2.58)
<30	694 (1.2)	44 (1.4)	1.05 (0.76–1.44)	15 (2.8)	1.83 (1.05–3.21)
Albuminuria					
No	51,319 (87.6)	2724 (84.6)	Ref	395 (71.6)	Ref
Microalbuminuria	5610 (9.6)	377 (11.7)	1.36 (1.21–1.54)	123 (22.3)	2.11 (1.69–2.63)
Macroalbuminuria	1635 (2.8)	118 (3.7)	1.37 (1.10–1.71)	34 (6.1)	1.90 (1.26–2.87)
Smoking	5240 (8.9)	289 (9.0)	0.93 (0.81–1.07)	54 (9.8)	1.12 (0.82–1.53)
Type of GLP-1 receptor agonist					
No GLP-1 receptor agonist	55,344 (94.5)	–	–	427 (77.4)	–
Dulaglutide	444 (<1)	444 (13.8)	–	21 (3.8)	–
Exenatide	0 (0)	0 (0.0)	–	0 (0)	–
Liraglutide	327 (<1)	327 (10.2)	–	18 (3.3)	–
Lixisenatide	<3 (<1)	<3 (0.1)	–	<3 (<1)	–
Semaglutide	2447 (4.2)	2447 (76.0)	–	85 (15.4)	–
Type of SGLT2 inhibitor					
No SGLT2 inhibitor	58,012 (99.1)	3095 (96.1)	–	–	–
Canagliflozin	8 (<1)	<3 (<1)	–	8 (1.4)	–
Dapagliflozin	309 (<1)	53 (1.6)	–	309 (56.0)	–
Empagliflozin	235 (<1)	70 (2.2)	–	235 (42.6)	–
Ertugliflozin	0 (0.0)	0 (0)	–	0 (0.0)	–

Odds ratios were calculated separately for each variable using age- and sex-adjusted logistic regression models. Values are presented as n (%).

a Insulin pump use was added post-hoc and was not included in the multiple imputation model. Missing values (26.1%) were assumed to be non-users.

To assess patient characteristics associated with use of GLP-1 receptor agonists or SGLT2 inhibitors, we conducted separate analyses for each variable, using logistic regression models adjusted for age as a categorical variable and sex. We restricted this analysis to study year 2024 to reflect contemporary clinical practice. Definition of variables used in this analysis for describing patient characteristics are provided in Supplementary Table S2.

To describe the incidence of diabetic ketoacidosis (defined by ICD-10 codes E100A, E101, E121, E131, E141 registered at any position in the National Patient Register) among patients with type 1 diabetes who initiated treatment with an SGLT2 inhibitor, we created a separate study cohort. In this analysis, we included all patients with type 1 diabetes (as defined previously) who filled their first prescription of an SGLT2 inhibitor during the study period. Patients were considered on-treatment for as long as prescriptions were refilled within the estimated duration of the most recent prescription, including a 90-day grace period. Number of days’ supply according to drug use are defined in Supplementary Table S1. Patients were followed from treatment initiation to outcome event (diabetic ketoacidosis), treatment discontinuation, death, emigration, or end of study period (31st December 2024).

### Additional analyses

We performed analyses of study drug use by subgroups according to status of cardiovascular disease, chronic kidney disease, and heart failure (definitions are provided in Supplementary Table S3). For liraglutide and semaglutide, we calculated the yearly prevalence by preparation indicated for obesity or diabetes; tirzepatide was not available in Sweden during the study period. We also used an intention-to-treat based exposure in the analyses of diabetic ketoacidosis among new users of an SGLT2 inhibitor.

### Sensitivity analyses

To assess the sensitivity of results to departures from the missing at random assumption, we conducted sensitivity analyses in which imputed values for individuals with missing BMI, eGFR and HbA1c were shifted by a constant Δ representing a hypothetical deviation from the missing at random assumption.^28^

### Role of the funding source

The funders had no role in the design and conduct of the study, collection, management, analysis, and interpretation of the data, preparation, review, or approval of the manuscript. The corresponding author had full access to all the data in the study and had final responsibility for the decision to submit for publication.

## Results

### Study population

The analyses included 72,698 patients with type 1 diabetes over the full study period (2007–2024). In 2024, 58,564 patients were included; median (IQR) age was 48 (33, 63) years and 44.2% were women. Overall, 20.6% had obesity, 9.4% had chronic kidney disease (including eGFR<60 or macroalbuminuria), 10.8% had cardiovascular disease, and 2.7% had heart failure (Table 1).

### Use of GLP-1 receptor agonists and SGLT2 inhibitors

**Fig. 1:**
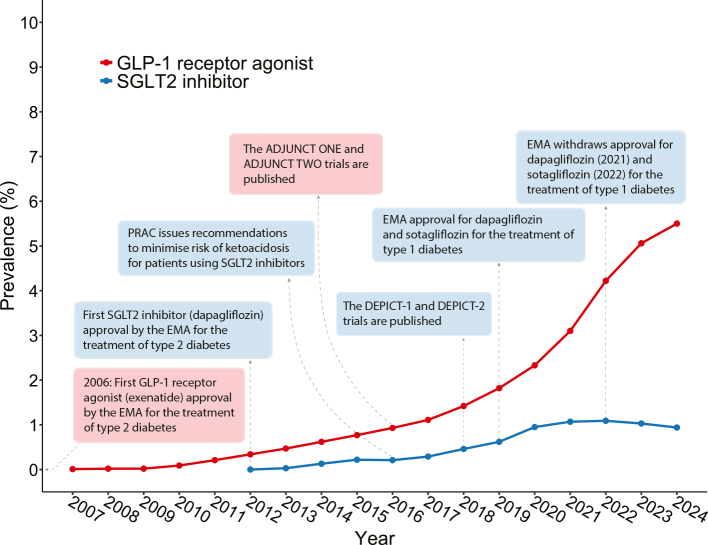
Yearly prevalence of GLP-1 receptor agonist and SGLT2 inhibitor use among adults with type 1 diabetes in Sweden, 2007–2024.

**Fig. 2:**
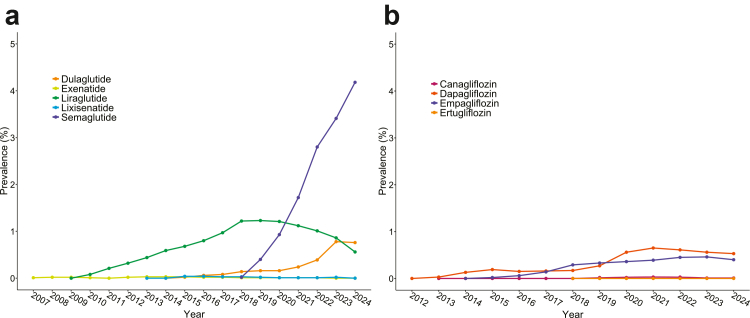
Yearly prevalence of use of individual (a) GLP-1 receptor agonists and (b) SGLT2 inhibitors, 2007–2024.

Use of GLP-1 receptor agonists increased continuously during the study period (0.01% in 2007 to 5.5% in 2024) (Fig. 1). Among those who used a GLP-1 receptor agonist in 2024, 56.5% were women, median (IQR) age was 51 (39, 61) years, and 64.3% had a BMI of ≥30 kg/m^2^ (Table 1). In 2016, the most common GLP-1 receptor agonist used was liraglutide (86.4%) whereas semaglutide was the most common in 2024 (76.0%) (Fig. 2). Supplementary Fig. S1 shows the yearly prevalence of use for specific GLP-1 receptor agonist indicated for obesity or diabetes.

Use of SGLT2 inhibitors increased from 0.03% in 2013 (the first full year following EMA approval in November 2012) to a peak of 1.1% in 2022, then decreased to 0.9% in 2024 (Fig. 1). Among those who used a SGLT2 inhibitor in 2024, 42.2% were women, median (IQR) age was 64 (53, 74) years, and 40.9% had a BMI of ≥30 kg/m^2^ (Table 1). Throughout the study period, dapagliflozin was the most commonly used SGLT2 inhibitor (71.7% in 2016 and 56.0% in 2024), although the proportion of empagliflozin increased from 27.3% in 2016 to 42.6% in 2024 (Fig. 2).

### Obesity and HbA1c category prevalence in patients without treatment with GLP-1 receptor agonists and SGLT2 inhibitors

**Fig. 3:**
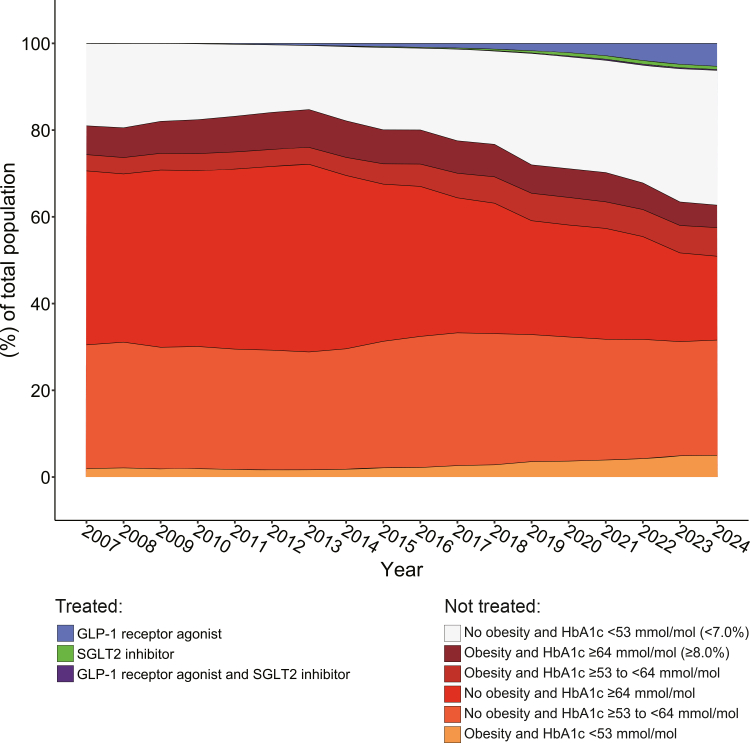
Proportions of the study population in 2024 by use of GLP-1 receptor agonist, SGLT2 inhibitor, or both, with patients using neither drug (not treated) further categorised by joint categories of obesity (BMI ≥30 kg/m^2^) and HbA1c level.

Fig. 3 shows the prevalence of joint categories of obesity and HbA1c categories in patients without treatment with GLP-1 receptor agonists and SGLT2 inhibitors. In 2024, of the total study population, 62.7% had obesity or an HbA1c of ≥53 mmol/mol (≥7.0%), 24.5% had an HbA1c of ≥64 mmol/mol (≥8.0%), 57.7% had an HbA1c of ≥53 mmol/mol, and 16.8% had obesity, and were not treated with a GLP-1 receptor agonist or an SGLT2 inhibitor (Table 1, Fig. 3, Supplementary Table S4).

### Subgroup analyses according to status of cardiovascular disease, and chronic kidney disease

**Fig. 4:**
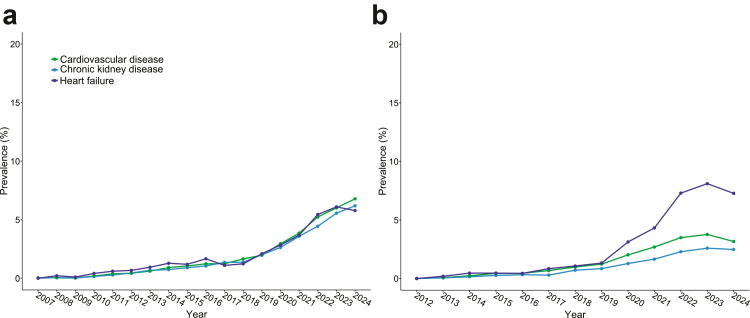
Yearly prevalence of (a) GLP-1 receptor agonist and (b) SGLT2 inhibitor use by status of cardiovascular disease, chronic kidney disease, and heart failure, 2007–2024.

Fig. 4 shows the use of GLP-1 receptor agonists and SGLT2 inhibitors across status of cardiovascular disease, chronic kidney disease and heart failure. Use of both drug classes increased until 2023 in all investigated subgroups, after which a slight decrease in use was observed among patients with heart failure (GLP-1 receptor agonists and SGLT2 inhibitors), chronic kidney disease (SGLT2 inhibitors only), and cardiovascular disease (SGLT2 inhibitors only). In 2024, 6.8% of patients with cardiovascular disease, 6.2% of patients with chronic kidney disease and 5.8% of patients with heart failure used a GLP-1 receptor agonist and 7.3% of patients with heart failure, 3.2% of patients with cardiovascular disease, 2.5% of patients with chronic kidney disease used an SGLT2 inhibitor (Supplementary Table S5).

### Patient characteristics associated with use of GLP-1 receptor agonists

Sociodemographic factors associated with use of a GLP-1 receptor agonist included female sex (OR 1.73 [95% CI 1.61–1.86]), place of birth outside Europe (OR 1.29 [1.11–1.51] vs. in Nordic countries), living with partner (OR 1.14 [1.06–1.23]), and age, peaking at 50–59 years (OR 6.40 [4.41–9.73] vs. <20 years). No statistically significant association was found with income while there was a negative association for higher education (medium or long tertiary education vs. primary or high school education, OR 0.69 [0.63–0.76]). Diabetes and comorbidity variables associated with GLP-1 receptor agonist use included higher BMI (BMI ≥35 vs. BMI <25 kg/m^2^, OR 33.80 [25.63–44.58]), higher HbA1c levels (HbA1c ≥ 75 mmol/mol (≥9.0%) vs. ≤48 mmol/mol (≤6.5%), OR 1.96 [1.67–2.30]), a history of ischemic heart disease and coronary revascularization (OR 1.64 [1.43–1.88]), heart failure (OR 1.32 [1.05–1.63]), and chronic kidney disease (OR 1.20 [1.06–1.37]). Moreover, use of other antidiabetic drugs (metformin, DPP4 inhibitors, sulfonylureas, other non-insulin diabetes drugs) were also associated with GLP-1 receptor agonist use (Table 1).

### Patient characteristics associated with use of SGLT2 inhibitors

Sociodemographic factors associated with SGLT2 inhibitor use included place of birth outside Europe (OR 2.11 [95% CI 1.52–2.94] vs. in Nordic countries), and older age, peaking at ≥70 years (OR 12.96 [4.93–52.47] vs. <20 years), while there was a negative association for female sex (OR 0.88 [0.74–1.04]) although not statistically significant. Higher individual and household income (high vs. low individual income, OR 0.72 [0.56–0.91]), and higher education were inversely associated with SGLT2 inhibitor use (medium or long tertiary education vs. primary or high school education, OR 0.59 [0.46–0.75]). Diabetes and comorbidity variables associated with SGLT2 inhibitor use included higher BMI (BMI ≥35 vs. BMI <25 kg/m^2^, OR 5.22 [3.71–7.33]), higher HbA1c levels (HbA1c ≥ 75 mmol/mol (≥9.0%) vs. ≤48 mmol/mol (≤6.5%), OR 1.99 [1.42–2.78]), history of ischemic heart disease and coronary revascularization (OR 3.10 [2.53–3.79]), heart failure (OR 5.81 [4.63–7.25]), and chronic kidney disease (OR 1.90 [1.52–2.37]). Use of other antidiabetic drugs (metformin, DPP-4 inhibitors, sulfonylureas, other non-insulin diabetes drugs) were also associated with SGLT2 inhibitor use (Table 1).

### Incidence of diabetic ketoacidosis among initiators of SGLT2 inhibitors

**Fig. 5:**
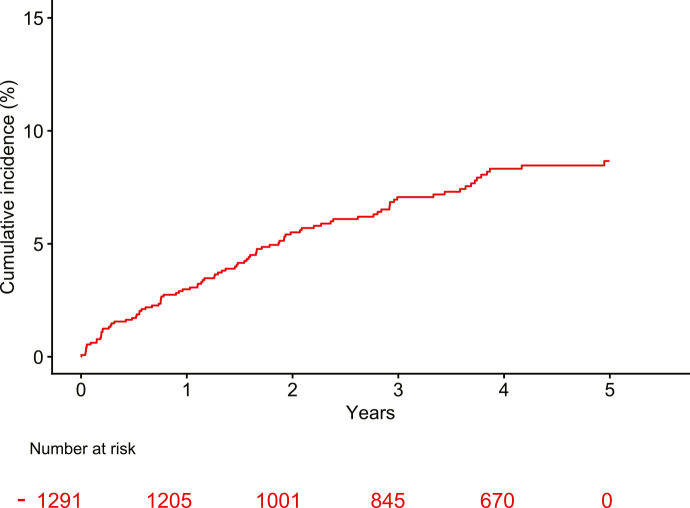
On-treatment cumulative incidence of diabetic ketoacidosis among new users of SGLT2 inhibitors.

Among 1291 new users of SGLT2 inhibitors, 53.6% were men and median (IQR) age was 57 (46, 69) years (Supplementary Table S6). Median (IQR) follow-up time on treatment was 414 (187, 922) days. During follow-up, 55 diabetic ketoacidosis events occurred (incidence rate 24.9, [95% CI 18.4–31.5] per 1000 person-years) (Fig. 5). Using an intention-to-treat based exposure (which also includes person-time occurring after treatment discontinuation), median (IQR) follow-up was 1509 (798, 1825) days and 94 diabetic ketoacidosis events occurred (incidence rate 20.4, [95% CI 16.3–24.5] per 1000 person-years) (Supplementary Fig. S2).

### Sensitivity analyses

In sensitivity analyses assessing the missing-at-random assumption underlying the multiple imputation of missing BMI, eGFR and HbA1c, the age- and sex-adjusted associations for use of GLP-1 receptor agonists and SGLT2 inhibitors, as well as the estimated proportion of untreated patients across joint categories of obesity and HbA1c were similar to those in the primary analyses across the range of Δ values examined (Supplementary Tables S7 and S8).

## Discussion

In this nationwide study including 72,698 patients with type 1 diabetes in Sweden between 2007 and 2024, use of GLP-1 receptor agonists and SGLT2 inhibitors had increased over time but remained low. In 2024, 5.5% of the patients had filled a prescription for a GLP-1 receptor agonist and 0.9% had filled a prescription for an SGLT2 inhibitor. In the same year, 24.5% of the type 1 diabetes patients had an HbA1c of ≥64 mmol/mol (≥8.0%) and 16.8% of the patients had obesity (BMI ≥30 kg/m^2^) and were not treated with either drug class. For both drug classes, use was associated with older age, obesity, poor glucose control, chronic kidney disease and cardiovascular comorbidities, although SGLT2 inhibitor use showed stronger associations with chronic kidney disease and cardiovascular comorbidities. The incidence of diabetic ketoacidosis among SGLT2 inhibitor users was 24.9 events per 1000 person-years. Given the clinical trials showing improvements in glucose control and weight reduction for GLP-1 receptor agonists and SGLT2 inhibitors in type 1 diabetes, potential may remain for improving patients' cardiometabolic risk profiles. However, whether expanded prescribing would be safe and beneficial across patient subgroups in routine clinical practice warrants further study, particularly with respect to the elevated diabetic ketoacidosis risk associated with SGLT2 inhibitors.

A study from the US,^16^ including analyses of around 405,019 patients with type 1 diabetes in 2023, found that 6.6% of the patients had been prescribed a GLP-1 receptor agonist and 2.4% of the patients had been prescribed an SGLT2 inhibitor, with the use of both drugs having increased continuously between 2010 and 2023. The low use of both drug classes in the US and in our study likely reflects the absence of regulatory approval for type 1 diabetes by the EMA and FDA. In Sweden, GLP-1 receptor agonists are reimbursed only for type 2 diabetes, with no reimbursement for obesity or type 1 diabetes. However, prior to 2023, some ambiguity in this policy may have allowed type 1 diabetes patients to receive reimbursement.^29^ For SGLT2 inhibitors, EMA withdrew the indications for type 1 diabetes for dapagliflozin in 2021^8^ and for sotagliflozin in 2022.^9^ This regulatory change likely contributed to the decline in SGLT2 inhibitor use observed in our study after 2022. The higher use of both drug classes in the US may partly reflect differences in healthcare reimbursement and prescribing practices. In both our and the US study,^16^ use of GLP-1 receptor agonists increased sharply between 2018 and 2024, potentially reflecting the improved glycaemic control and weight loss benefits observed with these drugs in clinical trials presented during this period.^1^, ^2^, ^3^

For both drug classes, use – based on models adjusted only for age and sex - was associated with older age, higher BMI, higher HbA1c levels, cardiovascular comorbidities, and socioeconomic factors including education and place of birth, and for SGLT2 inhibitors also income. The strongest predictor of GLP-1 receptor agonist use was BMI (e.g. adjusted OR for BMI ≥35 vs. <25 kg/m^2^: 33.80 (95% CI 25.63–44.58)), consistent with prescribing for the weight-reducing effects of these drugs. The inverse socioeconomic associations may potentially reflect poorer glycaemic control and a greater burden of comorbidity among individuals with lower socioeconomic status, differences in prescribing patterns across clinics serving populations of varying socioeconomic profiles, differential access to or follow-up in specialist care, or differences in patient or clinician attitudes towards unapproved drugs across patient socioeconomic status.

Clinical trials of SGLT2 inhibitors have shown improvement in glycaemic control, reduction of insulin requirements and promotion of weight loss in patients with type 1 diabetes.^4^ For example, a review including 8 clinical trials, reported that 24–26 weeks of SGLT2 inhibitor treatment reduced HbA1c levels by 2.7–5.9 mmol/mol (0.25–0.54%) from a baseline of 59.6–69.4 mmol/mol (7.6–8.5%), and reduced weight by 3–5% from a baseline of 78.7–87.3 kg. However, an approximately three-fold increased risk of diabetic ketoacidosis has damped enthusiasm regarding SGLT2 inhibitor use in this patient group.^30^ In our study, we observed an on-treatment incidence rate of 24.9 events per 1000 person-years. This rate is lower than those observed in clinical trials of SGLT2 inhibitors in type 1 diabetes, which have ranged from 31.0 to 59.4 events per 1000 person-years.^31^, ^32^, ^33^ A US claims-based study reported a higher diabetic ketoacidosis incidence than that observed in our study, ranging from 43 to 71 events per 1000 person-years, depending on the definition of type 1 diabetes.^34^ The lower incidence of diabetic ketoacidosis observed in our study could potentially be due to the universal health care system in Sweden providing structured care with yearly follow-up visits and risk management education for type 1 diabetes patients, and the high use rates of continuous glucose monitoring devices.^20^^,^^35^ Taken together our study along with previous evidence highlights diabetic ketoacidosis as an important safety concern when treating type 1 diabetes patients with SGLT2 inhibitors. Further studies may investigate the individual risk-benefit assessment for type 1 diabetes patients to guide treatment decisions and assess strategies for preventing diabetic ketoacidosis.

Use of GLP-1 receptor agonists in type 1 diabetes is less fraught with safety concerns. Clinical trials of GLP-1 receptor agonists in type 1 diabetes have shown benefits on glucose control and weight in type 1 diabetes.^1^, ^2^, ^3^ For example, in the ADJUST-T1D trial including 72 patients with obesity and type 1 diabetes with mean baseline HbA1c of 7.8% (62 mmol/mol) found that time in range for glucose increased by 8.8 percentage points and weight lowered by 8.8 kg for semaglutide vs. placebo at week 26 of follow-up.^1^ While statistically significant differences in safety outcomes were not observed in this trial, patients with type 1 diabetes receiving liraglutide in the larger ADJUNCT ONE and ADJUNCT TWO trials experienced an around 30% increased risk of hypoglycemia and a two- to four-fold increased risk of hyperglycemia with ketosis.^1^, ^2^, ^3^

Importantly, many patients with type 1 diabetes share characteristics with those with type 2 diabetes (e.g., obesity and insulin resistance).^36^ Moreover, chronic kidney disease and heart failure are prevalent comorbidities in type 1 diabetes. It is plausible that the risk-benefit balance of SGLT2 inhibitor or GLP-1 receptor agonist use is different for these subgroups who could potentially gain similar cardiorenal benefits with these treatments as those observed in the large clinical trials for type 2 diabetes, chronic kidney disease and heart failure. Several ongoing randomised trials (clinicaltrials.gov: NCT06082063; NCT06217302; NCT05822609) will provide more evidence. Moreover, it is assumed that improved glucose control, which these drugs confer in type 1 diabetes, reduces the risk of cardiovascular and renal complications.^37^

Our analyses show that overall use of GLP-1 receptor agonists and SGLT2 inhibitors among type 1 diabetes patients remains relatively low. Specifically, 24.5% of the patients had an HbA1c of ≥64 mmol/mol (≥8.0%) and 16.8% of the patients had obesity (BMI of ≥30 kg/m^2^) and were not treated with either drug class. These findings suggest that many patients with type 1 diabetes could potentially improve their cardiometabolic risk profiles through treatment with GLP-1 receptor agonists and potentially SGLT2 inhibitors. However, regulatory approval for this indication is currently lacking and the balance of risks and benefits across clinically relevant subgroups remains incompletely understood. In particular, safe and effective treatment strategies need to be investigated, for example by identifying patients at increased risk and employing risk mitigation strategies for diabetic ketoacidosis (e.g., continuous ketone measurement, or dose reductions).

Strengths of this study include the use of a large, unselected national study population from routine clinical practice and the use of nationwide registers with detailed data on prescription drug use, diagnoses registered during hospitalization and outpatient visits, clinical variables such as BMI and HbA1c, as well as markers of socioeconomic status. Moreover, our study includes near complete coverage of the time period when GLP-1 receptor agonists and SGLT2 inhibitor have been available.

Our study is not without limitations. First, although our inclusion criteria aimed to minimize the inclusion of patients with diabetes types other than type 1 diabetes, misclassification may have occurred due to lack of data on c-peptide levels and islet auto-antibody titres. In 2024, 2870 (4.9%) of patients in our study population had used metformin, with this proportion being larger in users of GLP-1 receptor agonists and SGLT2 inhibitors. Although this could indicate some degree of misclassification of diabetes type, it may also reflect clinical decisions for patients with type 1 diabetes and insulin resistance, as metformin could be used as an adjunct therapy in this group despite conflicting and uncertain trial evidence on its potential benefits.^38^^,^^39^ Second, we did not analyze lower BMI categories comprising overweight (25 to <30 kg/m^2^), although weight reduction among those with a BMI in this range may also lead to improved outcomes. Third, while we had complete coverage of data on filled prescriptions, information about actual drug intake and clinical rationale for prescribing these medications was not available. Fourth, as our study was performed in Sweden where universal health care is provided, our findings may not be applicable to countries with other forms of health care systems. Fifth, as drug use was defined as having filled at least one prescription during the study year, we did not assess the proportion of sustained vs. transient users. Lastly, although diagnoses registered in the Swedish Patient Register have high sensitivity and positive predictive values,^21^ our findings might be affected by misclassification of diagnostic codes used for defining patient characteristics.

In a national population of patients with type 1 diabetes, use of GLP-1 receptor agonists and SGLT2 inhibitors has increased over time but remains relatively low at 5.5% for GLP-1 receptor agonists and 0.9% for SGLT2 inhibitors in 2024. It is possible that cardiometabolic risk factors among type 1 diabetes patients could be improved using ancillary treatment with SGLT2 inhibitors and GLP-1 receptor agonists. However, regulatory approval for this indication is currently lacking and the balance of risks and benefits across clinically relevant subgroups remains incompletely understood. In particular, strategies for safe use need to be investigated, especially with respect to the diabetic ketoacidosis risk associated with SGLT2 inhibitors.

## Contributors

CEL, BP, LP and PU conceived and designed the study. All authors contributed to acquisition, analysis or interpretation of data. CEL conducted the statistical analyses. CEL and PU drafted the manuscript, and all authors critically revised it for important intellectual content. BP and PU obtained funding, and BP, LP and PU supervised the study. CEL and PU had full access to all the data in the study and take responsibility for the integrity of the data and the accuracy of the data analysis. All authors were responsible for the decision to submit the manuscript.

## Data sharing statement

No additional data available. The data analysed in this study were based on Swedish nationwide registers. Individual level data from the registers can only be accessed through secure servers and only export of aggregated data, as presented in research articles, is allowed. Permission to access data can be made only after fulfilling specific requirements to safeguard the anonymity of the study participants and other data safety issues. For these reasons, data cannot be made generally available.

## Declaration of interests

All authors have completed the Unified Competing Interest form at www.icmje.org/coi_disclosure.pdf (available on request from the corresponding author) and have the following declarations. BE reports personal fees from Amgen, AstraZeneca, Boerhringer Ingelheim, Eli Lilly, Merck Sharp & Dohme, Mundipharma, Navamedic, Novo Nordisk, and RLS Global outside the submitted work, and grants from Sanofi outside the submitted work. CEL, LP, BP, PU report no conflict of interest.
