# Supplementary material for: Use of GLP-1 Receptor Agonists and SGLT2 Inhibitors Among Patients with Type 1 Diabetes: A Nationwide Register-Based Cohort Study
Source: Lancet Reg Health Eur. 2026 Jun 4;67:101729. doi: 10.1016/j.lanepe.2026.101729 (PMC13265897; doi:10.1016/j.lanepe.2026.101729)

**SUPPLEMENTARY MATERIAL**

Lim et al. Use of GLP-1 receptor agonists and SGLT2 inhibitors in a national population of patients with type 1 diabetes.

Table of Contents

[**Supplementary Table 1** ATC codes and estimates days of supply for study drugs. 3](#_Toc230608742)

[**Supplementary Table 2** Definition of variables used to assess factors associated with the likelihood of receiving a GLP-1 receptor agonist or an SGLT2 inhibitor and for describing patient characteristics. 4](#_Toc230608743)

[**Supplementary Table 3** Definitions for cardiovascular disease, chronic kidney disease, and heart failure. 6](#_Toc230608744)

[**Supplementary Table 4** Proportion of study population treated with GLP-1 receptor agonists or SGLT2 inhibitors and untreated patients by obesity and HbA1c status in 2024. 7](#_Toc230608745)

[**Supplementary Table 5** Proportion of patients treated according to status of cardiovascular disease, chronic kidney disease and heart failure in 2024. 8](#_Toc230608746)

[**Supplementary Table 6** Patient characteristics among new users of SGLT2 inhibitor. Values are presented as n (%). 9](#_Toc230608747)

[**Supplementary Table 7** Age- and sex-adjusted odds ratios for use of GLP-1 receptor agonist and SGLT2 inhibitor in 2024 by BMI, eGFR, and HbA1c category in sensitivity analyses assessing the missing at random (MAR) assumption. Δ denotes the departure applied to imputed values for individuals with missing data. The Δ = 0 column corresponds to the imputed values generated under the MAR assumption. 13](#_Toc230608748)

[**Supplementary Table 8** Proportion of total study population not treated with GLP-1 receptor agonists or SGLT2 inhibitors in 2024 by joint obesity and HbA1c categories in sensitivity analyses assessing the missing at random (MAR) assumption. Δ denotes the departure applied to imputed values for individuals with missing data. The Δ = 0 column corresponds to the imputed values generated under the MAR assumption. 15](#_Toc230608749)

[**Supplementary Figure 1** Annual prevalence of specific GLP-1 receptor agonist by preparation indicated for diabetes or obesity. 16](#_Toc230608750)

[**Supplementary Figure 2** Cumulative incidence of diabetic ketoacidosis events among new users of SGLT2 inhibitors using an intention-to-treat based exposure. 17](#_Toc230608751)

# **Supplementary Table 1** ATC codes and estimates days of supply for study drugs.

|  | **ATC codes** | **Estimated days of supply** |
| --- | --- | --- |
| **SGLT2 inhibitors** | Dapagliflozin |  |
|  | A10BK01 | 1.0 per tablet |
|  | A10BD15 | 0.5 per tablet |
|  | A10BD21 | 1.0 per tablet |
|  | Canagliflozin |  |
|  | A10BK02 | 1.0 per tablet |
|  | A10BD16 | 0.5 per tablet |
|  | Empagliflozin |  |
|  | A10BK03 | 1.0 per tablet |
|  | A10BD19 | 1.0 per tablet |
|  | A10BD20 | 0.5 per tablet |
|  | Ertugliflozin |  |
|  | A10BK04 | 1.0 per tablet |
|  | A10BD23 | 0.5 per tablet |
|  | A10BD24 | 1.0 per tablet |
| **GLP-1 receptor agonists** | Exenatide |  |
|  | A10BJ01 |  |
|  | Liraglutide |  |
|  | A10BJ02 |  |
|  | A10AE56 |  |
|  | Lixisenatide |  |
|  | A10BJ03 |  |
|  | A10AE54 |  |
|  | Dulaglutide |  |
|  | A10BJ05 |  |
|  | Semaglutide |  |
|  | A10BJ06 |  |

# **Supplementary Table 2** Definition of variables used to assess factors associated with the likelihood of receiving a GLP-1 receptor agonist or an SGLT2 inhibitor and for describing patient characteristics.

| **Sociodemographic characteristics** | **ICD/categories** |
| --- | --- |
| Sex | Women; men |
| Age (years) | <20, 20-29, 30-39, 40-49, 50-59, 60-69, ≥70 |
| Place of birth | Nordic countries (Sweden, Norway, Denmark, Finland); Rest of Europe; Outside Europe |
| Education | Primary school and high school; vocational or short-term tertiary education; medium or long-term tertiary education |
| Individual income | Income levels were categorized into quartiles within both sex and each age group as specified above. For example, if a male patient is 45 years old, the patient is categorized to high income if they are in the 4^th^ quartile of income among males ages 40-49.  High – 4^th^ quartile Medium – 2^nd^ and 3^rd^ quartile Low – 1^st^ quartile |
| Household income | Total household income weighted by household composition.^17^ Categorized into levels of high, medium and low income as above. |
| Living with partner | Yes; no |
| **Medical history  (10-year look-back)** | *ICD-10 code and procedure code* |
| Ischaemic heart disease and coronary revascularization | ICD-10: I20-I25; procedure FNA, FNB, FNC, FND, FNE, FNG, FNP02, FNP12, FNQ05, FNQ12, FNR22 |
| Ischaemic stroke | ICD-10: I63, I693 |
| Arterial disease (including amputation) | ICD-10: I65, I70, I72, I73, I74, I77, K550, K551, E105, E135, E145; procedure code: NFQ, NGQ, NHQ |
| Atrial fibrillation | ICD-10: I48 |
| Heart failure | ICD-10: I110, I130, I132, I50 |
| Chronic kidney disease | eGFR <60 ml/min/1.73 m^2^ or macroalbuminuria or ICD-10: Z49, Z940, Z992; procedure code: KAS, DR012, DR013, DR014, DR015, DR016, DR023, DR024, DR055, DR056, DR060, DR061 |
| Liver disease | ICD-10: B15-B19, K70-K77, C22 |
| Pancreatitis | ICD-10: K85 |
| Ketoacidosis | ICD-10: E100A, E101, E121, E131, E141 |
| Diabetic eye complications | ICD-10: E103, E133, E143 H280, H358, H360  Procedure code: CKC12, CKD65 |
| Other diabetic complications | ICD-10: E100 (excl E100A), E130, E140, E160, E161, E162, G990, G590, G632, E134, E136, E137, E138, E144, E146, E147, E148, M142, M146, M908, L984 |
| Psychiatric disorder | ICD-10: F00-F09, F20- F99 |
| Mental and behavioral disorders due to psychoactive substance use | ICD-10: F10-F19 |
| **Medications in the last 12 months** | *ATC code* |
| SGLT2 inhibitor**^a^** | Dapagliflozin: A10BK01, A10BD15, A10BD21, *A10BD25*; Canagliflozin: A10BK02, A10BD16; Empagliflozin: A10BK03, A10BD19, A10BD20, *A10BD27*; Ertugliflozin: A10BK04, A10BD23, A10BD24; Ipragliflozin: *A10BK05*; Sotagliflozin: A10BK06; Luseogliflozin: *A10BK07* |
| GLP-1 receptor agonist**^a^** | Exenatide: A10BJ01; Liraglutide: A10BJ02, A10AE56; Lixisenatide: A10BJ03, A10AE54; *Albiglutide:* *A10BJ04;* Dulaglutide: A10BJ05; Semaglutide: A10BJ06; *Beinaglutide:* *A10BJ07* |
| Insulin**^a^** | *A10AA*, A10AB, A10AC, A10AD, A10AE, *A10AF* |
| Metformin | A10BA02, *A10BD02*, *A10BD03*, A10BD05, A10BD07, A10BD08, A10BD10, A10BD11, A10BD13, *A10BD14*, A10BD15, A10BD16, *A10BD17*, *A10BD18*, A10BD20, *A10BD22*, A10BD23, *A10BD25*, *A10BD26*, *A10BD27* |
| DPP4 inhibitors | A10BH, A10BD07, A10BD08, A10BD09, A10BD10, A10BD11, *A10BD12*, A10BD13, *A10BD18*, A10BD19, A10BD21, *A10BD22*, A10BD24, *A10BD25*, *A10BD27* |
| Sulfonylureas | A10BB, A10BD01, A10BD02, A10BD04, A10BD06 |
| Other non-insulin diabetes drugs (glitazones, glinides, acarbose) | Glitazones, glinides, acarbose: A10BF, A10BG, *A10BD03*, *A10BD04*, A10BD05, A10BD06, A10BD09, *A10BD12,* *A10BD14*, *A10BD17*, *A10BD26*, A10BX |
| Time since first diabetes drug (ever look-back) | <3 year, ≥3 to <10 years, ≥10 years |
| Number of diabetes drugs^b^ | 0; 1 or 2, ≥3 |
| Platelet inhibitors^c^ | B01AC24, B01AC04, B01AC22 |
| Statins^c^ | C10AA, C10B |
| ACE inhibitors/Angiotensin II receptor blockers^c^ | C09A, C09B, C09C, C09D |
| Calcium antagonists^c^ | C08C, C08D, C09BB, C09DB, C09DX01 |
| β‑blockers^c^ | C07 |
| Diuretics | C03C, C03EB, C03A, C03B, C03D, C03EA |
| **National Diabetes Register variables (1-year look back)** |  |
| Blood pressure | Normotension: SBP <140 mmHg AND DBP <90 mmHg  Stage 1 hypertension: SBP ≥140 to <160 mmHg or DBP: ≥90 to <100mmHg  Stage 2 hypertension: SBP ≥160 mmHg or DBP: ≥100 mmHg |
| HbA1c | ≤48 mmol/mol (≤6.5%) 49-52 mmol/mol (6.6-6.9%) 53-63 mmol/mol (7.0-7.9%) 64-74 mmol/mol (8.0 – 8.9%) ≥75 mmol/mol (≥9.0%) |
| Body mass index | Normal weight: <25 kg/m^2^ Overweight: ≥25 to <30 kg/m^2^ Obese class I: ≥30 to <35 kg/m^2^ Obese class II/III: ≥35 kg/m^2^ |
| eGFR (ml/min per 1.73m^2^) | ≥90; 60 to <90; 30 to <60; <30 |
| Albuminuria | Macroalbuminuria; microalbuminuria; none |
| Smoking | Yes/No |

# **Supplementary Table 3** Definitions for cardiovascular disease, chronic kidney disease, and heart failure.

|  | **ICD 10/procedure code** |
| --- | --- |
| Cardiovascular disease | Coronary revascularization: procedure code: FNA, FNB, FNC, FND, FNE, FNG, FNP02, FNP12, FNQ05, FNQ12, FNR22  Ischaemic heart disease: I20-I25  Ischaemic stroke: I63, I69.3  Arterial disease, including amputation: I65, I70, I72, I73, I74, I77, K550, K551, E105, E135, E145; procedure code: NFQ, NGQ, NHQ |
| Chronic kidney disease | eGFR <60 ml/min/1.73 m^2^ or macroalbuminuria or ICD-10: Z49, Z940, Z992; procedure code: KAS, DR012, DR013, DR014, DR015, DR016, DR023, DR024, DR055, DR056, DR060, DR061 |
| Heart failure | I110, I130, I132, I50 |

# **Supplementary Table 4** Proportion of study population treated with GLP-1 receptor agonists or SGLT2 inhibitors and untreated patients by obesity and HbA1c status in 2024.

|  | **Proportion of study population (%)** |
| --- | --- |
| **Treated** |  |
| GLP-1 receptor agonist | 5.3 |
| SGLT2 inhibitor | 0.7 |
| GLP-1 receptor agonist and SGLT2 inhibitor | 0.2 |
| **Not treated** |  |
| Obesity (BMI ≥30 kg/m^2^) and HbA1c ≥64 mmol/mol (≥8.0%) | 5.2 |
| Obesity and HbA1c ≥53 to <64 mmol/mol (≥7.0% to 8.0%) | 6.6 |
| Obesity and HbA1c <53 mmol/mol | 5.0 |
| No obesity and HbA1c ≥64 mmol/mol | 19.3 |
| No obesity and HbA1c ≥53 to <64 mmol/mol | 26.6 |
| No obesity and HbA1c <53 mmol/mol | 31.1 |

# **Supplementary Table 5** Proportion of patients treated according to status of cardiovascular disease, chronic kidney disease and heart failure in 2024.

| **Subgroup** | **GLP-1 receptor agonist (%)** | **SGLT2 inhibitor (%)** |
| --- | --- | --- |
| Cardiovascular disease | 6.8 | 3.2 |
| Chronic kidney disease | 6.2 | 2.5 |
| Heart failure | 5.8 | 7.3 |

# **Supplementary Table 6** Patient characteristics among new users of SGLT2 inhibitor. Values are presented as n (%).

| **Characteristic** | **N = 1291** |
| --- | --- |
| **Type of SGLT2 inhibitor** |  |
| *Canagliflozin* | 19 (1.5) |
| *Dapagliflozin* | 783 (60.7) |
| *Empagliflozin* | 488 (37.8) |
| *Ertugliflozin* | <3 (0.1) |
| **Sociodemographic characteristics** |  |
| **Men** | 692 (53.6) |
| **Age** |  |
| <20 | 7 (0.5) |
| 20-29 | 84 (6.5) |
| 30-39 | 117 (9.1) |
| 40-49 | 201 (15.6) |
| 50-59 | 300 (23.2) |
| 60-69 | 287 (22.2) |
| ≥70 | 295 (22.9) |
| **Place of birth** |  |
| *Nordic countries* | 1152 (89.2) |
| *Outside Europe* | 84 (6.5) |
| *Rest of Europe* | 55 (4.3) |
| **Education** |  |
| *Primary school and high school* | 935 (72.4) |
| *Vocational or short-term tertiary education* | 156 (12.1) |
| *Medium or long tertiary education* | 188 (14.6) |
| *Unknown* | 12 (0.9) |
| **Income** |  |
| *Low income* | 383 (29.7) |
| *Middle income* | 544 (42.1) |
| *High income* | 364 (28.2) |
| **Household income** |  |
| *Low income* | 275 (21.3) |
| *Middle income* | 550 (42.6) |
| *High income* | 466 (36.1) |
| **Living with partner** | 594 (45.6) |
| **Medical history (10-year look-back)** |  |
| **Cardiovascular disease** | 456 (35.3) |
| **Ischemic heart disease and coronary revascularization** | 348 (27.0) |
| **Ischemic stroke** | 71 (5.5) |
| **Arterial disease (including amputation)** | 204 (15.8) |
| **Atrial fibrillation** | 165 (12.8) |
| **Heart failure** | 261 (20.2) |
| **Chronic kidney disease** |  |
| *No* | 606 (46.9) |
| *Yes* | 210 (16.3) |
| *Unknown* | 475 (36.8) |
| **Liver disease** | 32 (2.5) |
| **Pancreatitis** | 17 (1.3) |
| **Ketoacidosis** | 105 (8.1) |
| **Diabetic eye complications** | 922 (71.4) |
| **Other diabetic complications** | 291 (22.5) |
| **Psychiatric disorder** | 240 (18.6) |
| **Mental and behavioral disorders due to psychoactive substance use** | 91 (7.0) |
| **Medications in the previous year** |  |
| **GLP-1 receptor agonist** | 220 (17.0) |
| **Insulin pump use** | 166 (12.9) |
| **Metformin** | 348 (27.0) |
| **DPP4 inhibitors** | 64 (5.0) |
| **Sulfonylureas** | 14 (1.1) |
| **Other non-insulin diabetes drugs (glitazones, glinides, acarbose)** | 12 (0.9) |
| **Platelet inhibitors** | 115 (8.9) |
| **Statins** | 899 (69.6) |
| **ACE inhibitors/Angiotensin II receptor blockers** | 840 (65.1) |
| **Calcium antagonists** | 406 (31.4) |
| **Beta-blockers** | 540 (41.8) |
| **Diuretics** | 430 (33.3) |
| **Time since first diabetes drug** |  |
| <3 year | 55 (4.3) |
| ≥3 to <10 years | 288 (22.3) |
| ≥10 years | 948 (73.4) |
| **Number of diabetes drugs** |  |
| *1 or 2* | 735 (56.9) |
| ≥3 | 556 (43.1) |
| **Blood pressure** |  |
| Normotension | 695 (53.8) |
| Stage 1 hypertension | 254 (19.7) |
| Stage 2 hypertension | 59 (4.6) |
| *Unknown* | 283 (21.9) |
| **HbA1c** |  |
| ≤48 mmol/mol (≤6.5%) | 70 (5.4) |
| 49-52 mmol/mol (6.6-6.9%) | 61 (4.7) |
| 53-63 mmol/mol (7.0-7.9%) | 311 (24.1) |
| 64-74 mmol/mol (8.0 – 8.9%) | 331 (25.6) |
| ≥75 mmol/mol (≥9.0%) | 305 (23.6) |
| *Unknown* | 213 (16.5) |
| **Body mass index** |  |
| Normal weight: <25 kg/m^2^ | 151 (11.7) |
| Overweight: ≥25 to <30 kg/m^2^ | 254 (19.7) |
| Obese class I: ≥30 to <35 kg/m^2^ | 296 (22.9) |
| Obese class II/III: ≥35 kg/m^2^ | 184 (14.3) |
| *Unknown* | 406 (31.4) |
| **eGFR (ml/min per 1.73m^2** |  |
| ≥90 | 384 (29.7) |
| 60 to <90 | 358 (27.7) |
| 30 to <60 | 163 (12.6) |
| <30 | 14 (1.1) |
| *Unknown* | 372 (28.8) |
| **Albuminuria** |  |
| No | 591 (45.8) |
| Microalbuminuria | 162 (12.5) |
| Macroalbuminuria | 59 (4.6) |
| *Unknown* | 479 (37.1) |
| **Smoking** |  |
| *0* | 894 (69.2) |
| *1* | 96 (7.4) |
| *Unknown* | 301 (23.3) |

# **Supplementary Table 7** Age- and sex-adjusted odds ratios for use of GLP-1 receptor agonist and SGLT2 inhibitor in 2024 by BMI, eGFR, and HbA1c category in sensitivity analyses assessing the missing at random (MAR) assumption. Δ denotes the departure applied to imputed values for individuals with missing data. The Δ = 0 column corresponds to the imputed values generated under the MAR assumption.

| **BMI (kg/m²)** | **Δ = −3** | **Δ = −2** | | **Δ = −1** | | **Δ = 0** | **Δ = +1** | | **Δ = +2** | | **Δ = +3** |
| --- | --- | --- | --- | --- | --- | --- | --- | --- | --- | --- | --- |
|  | OR (95% CI) | OR (95% CI) | | OR (95% CI) | | OR (95% CI) | OR (95% CI) | | OR (95% CI) | | OR (95% CI) |
| **Use of GLP-1 receptor agonist** |  |  | |  | |  |  | |  | |  |
| BMI (kg/m²) |  |  | |  | |  |  | |  | |  |
| Normal weight: <25 kg/m² | Ref | Ref | | Ref | | Ref | Ref | | Ref | | Ref |
| Overweight: ≥25 to <30 kg/m² | 4.26 (3.50−5.19) | 4.48 (3.66−5.47) | | 4.27 (3.53−5.16) | | 4.07 (3.38−4.90) | 3.79 (3.19−4.51) | | 3.45 (2.90−4.11) | | 2.97 (2.48−3.55) |
| Obese class I: ≥30 to <35 kg/m² | 13.01 (10.49−16.13) | 14.25 (11.48−17.68) | | 14.52 (11.77−17.90) | | 13.97 (11.35−17.20) | 13.21 (10.88−16.03) | | 11.56 (9.57−13.96) | | 9.49 (7.97−11.31) |
| Obese class II/III: ≥35 kg/m² | 27.06 (21.37−34.27) | 30.36 (23.22−39.69) | | 32.06 (24.53−41.90) | | 33.80 (26.06−43.85) | 33.73 (26.57−42.82) | | 31.41 (25.06−39.37) | | 26.60 (21.71−32.59) |
| **Use of SGLT2 inhibitor** |  |  | |  | |  |  | |  | |  |
| BMI (kg/m²) |  |  | |  | |  |  | |  | |  |
| Normal weight: <25 kg/m² | Ref | Ref | | Ref | | Ref | Ref | | Ref | | Ref |
| Overweight: ≥25 to <30 kg/m² | 1.73 (1.38−2.19) | 1.74 (1.37−2.21) | | 1.70 (1.35−2.13) | | 1.69 (1.31−2.17) | 1.61 (1.24−2.09) | | 1.50 (1.15−1.95) | | 1.47 (1.11−1.93) |
| Obese class I: ≥30 to <35 kg/m² | 2.91 (2.25−3.75) | 2.91 (2.25−3.76) | | 2.98 (2.29−3.86) | | 2.87 (2.20−3.73) | 2.76 (2.09−3.64) | | 2.55 (1.91−3.40) | | 2.39 (1.77−3.22) |
| Obese class II/III: ≥35 kg/m² | 5.42 (3.88−7.56) | 5.59 (3.98−7.84) | | 5.44 (3.91−7.57) | | 5.22 (3.73−7.31) | 4.96 (3.52−6.97) | | 4.49 (3.24−6.23) | | 4.19 (3.02−5.83) |
|  | | | | | | | | | | | |
| **eGFR (mL/min/1.73m²)** | Δ **= −15** | Δ **= −10** | | Δ **= −5** | | Δ **= 0** | Δ **= +5** | | Δ **= +10** | | Δ **= +15** |
|  | OR (95% CI) | OR (95% CI) | | OR (95% CI) | | OR (95% CI) | OR (95% CI) | | OR (95% CI) | | OR (95% CI) |
| **Use of GLP-1 receptor agonist** |  |  | |  | |  |  | |  | |  |
| eGFR (mL/min/1.73m²) |  |  | |  | |  |  | |  | |  |
| ≥90 mL/min/1.73m² | Ref | Ref | | Ref | | Ref | Ref | | Ref | | Ref |
| 60 to <90 mL/min/1.73m² | 0.88 (0.80−0.95) | 0.89 (0.81−0.96) | | 0.90 (0.83−0.99) | | 0.91 (0.83−0.99) | 0.92 (0.84−1.01) | | 0.93 (0.85−1.01) | | 0.96 (0.88−1.04) |
| 30 to <60 mL/min/1.73m² | 0.90 (0.78−1.03) | 0.97 (0.83−1.12) | | 1.03 (0.88−1.21) | | 1.07 (0.91−1.27) | 1.13 (0.96−1.33) | | 1.16 (0.98−1.37) | | 1.21 (1.02−1.43) |
| <30 mL/min/1.73m² | 0.96 (0.71−1.31) | 0.99 (0.71−1.38) | | 0.98 (0.71−1.36) | | 1.05 (0.76−1.44) | 1.04 (0.74−1.46) | | 0.98 (0.68−1.41) | | 1.01 (0.69−1.46) |
| Chronic kidney disease | 1.04 (0.93−1.17) | 1.11 (0.98−1.25) | | 1.16 (1.03−1.31) | | 1.20 (1.06−1.37) | 1.24 (1.08−1.41) | | 1.24 (1.09−1.42) | | 1.26 (1.10−1.44) |
| **Use of SGLT2 inhibitor** |  |  | |  | |  |  | |  | |  |
| eGFR (mL/min/1.73m²) |  |  | |  | |  |  | |  | |  |
| ≥90 mL/min/1.73m² | Ref | Ref | | Ref | | Ref | Ref | | Ref | | Ref |
| 60 to <90 mL/min/1.73m² | 1.16 (0.91−1.48) | 1.11 (0.88−1.40) | | 1.14 (0.90−1.43) | | 1.13 (0.89−1.42) | 1.09 (0.88−1.36) | | 1.10 (0.89−1.37) | | 1.08 (0.88−1.33) |
| 30 to <60 mL/min/1.73m² | 1.85 (1.40−2.45) | 1.89 (1.42−2.51) | | 2.03 (1.55−2.66) | | 1.97 (1.51−2.58) | 1.96 (1.48−2.60) | | 1.98 (1.52−2.59) | | 1.92 (1.46−2.51) |
| <30 mL/min/1.73m² | 1.98 (1.25−3.14) | 2.13 (1.31−3.44) | | 1.90 (1.11−3.24) | | 1.83 (1.05−3.21) | 1.84 (1.05−3.25) | | 1.66 (0.88−3.12) | | 1.64 (0.88−3.05) |
| Chronic kidney disease | 1.77 (1.44−2.18) | 1.87 (1.51−2.32) | | 1.91 (1.54−2.38) | | 1.90 (1.52−2.37) | 1.93 (1.55−2.40) | | 1.91 (1.53−2.38) | | 1.88 (1.51−2.34) |
|  | | | | | | | | | | | |
| **HbA1c (mmol/mol)** | Δ **= −10** | | Δ **= −5** | | Δ **= 0** | | | Δ **= +5** | | Δ **= +10** | |
|  | OR (95% CI) | | OR (95% CI) | | OR (95% CI) | | | OR (95% CI) | | OR (95% CI) | |
| **Use of GLP-1 receptor agonist** |  | |  | |  | | |  | |  | |
| HbA1c |  | |  | |  | | |  | |  | |
| ≤48 mmol/mol (≤6.5%) | Ref | | Ref | | Ref | | | Ref | | Ref | |
| 49–52 mmol/mol (6.6–6.9%) | 1.20 (1.03−1.39) | | 1.20 (1.05−1.38) | | 1.20 (1.04−1.39) | | | 1.18 (1.03−1.36) | | 1.17 (1.02−1.35) | |
| 53–63 mmol/mol (7.0–7.9%) | 1.41 (1.26−1.58) | | 1.41 (1.26−1.59) | | 1.42 (1.26−1.59) | | | 1.38 (1.24−1.55) | | 1.37 (1.22−1.53) | |
| 64–74 mmol/mol (8.0–8.9%) | 1.89 (1.66−2.15) | | 1.90 (1.67−2.16) | | 1.87 (1.64−2.13) | | | 1.80 (1.58−2.05) | | 1.71 (1.52−1.94) | |
| ≥75 mmol/mol (≥9.0%) | 2.02 (1.76−2.33) | | 2.01 (1.74−2.32) | | 1.96 (1.67−2.29) | | | 1.88 (1.62−2.18) | | 1.77 (1.53−2.06) | |
| **Use of SGLT2 inhibitor** |  | |  | |  | | |  | |  | |
| HbA1c |  | |  | |  | | |  | |  | |
| ≤48 mmol/mol (≤6.5%) | Ref | | Ref | | Ref | | | Ref | | Ref | |
| 49–52 mmol/mol (6.6–6.9%) | 1.28 (0.94−1.75) | | 1.29 (0.93−1.79) | | 1.35 (0.96−1.90) | | | 1.34 (0.94−1.90) | | 1.44 (1.01−2.04) | |
| 53–63 mmol/mol (7.0–7.9%) | 1.31 (1.03−1.68) | | 1.34 (1.03−1.73) | | 1.38 (1.03−1.84) | | | 1.40 (1.05−1.87) | | 1.44 (1.08−1.92) | |
| 64–74 mmol/mol (8.0–8.9%) | 1.63 (1.25−2.13) | | 1.67 (1.26−2.21) | | 1.77 (1.32−2.38) | | | 1.74 (1.29−2.34) | | 1.73 (1.26−2.38) | |
| ≥75 mmol/mol (≥9.0%) | 1.83 (1.32−2.53) | | 1.97 (1.42−2.74) | | 1.99 (1.42−2.78) | | | 2.03 (1.46−2.82) | | 2.08 (1.49−2.90) | |

# **Supplementary Table 8** Proportion of total study population not treated with GLP-1 receptor agonists or SGLT2 inhibitors in 2024 by joint obesity and HbA1c categories in sensitivity analyses assessing the missing at random (MAR) assumption. Δ denotes the departure applied to imputed values for individuals with missing data. The Δ = 0 column corresponds to the imputed values generated under the MAR assumption.

| **BMI (kg/m²)** | **Δ = −3** | **Δ = −2** | | **Δ = −1** | | **Δ = 0** | **Δ = +1** | | **Δ = +2** | | **Δ = +3** |
| --- | --- | --- | --- | --- | --- | --- | --- | --- | --- | --- | --- |
|  | Not treated, % | Not treated, % | | Not treated, % | | Not treated, % | Not treated, % | | Not treated, % | | Not treated, % |
| Obesity (BMI ≥30 kg/m^2^) and HbA1c ≥64 mmol/mol (≥8.0%) | 3.7 | 4.1 | | 4.6 | | 5.2 | 5.9 | | 6.8 | | 7.9 |
| Obesity and HbA1c ≥53 to <64 mmol/mol (≥7.0% to 8.0%) | 4.8 | 5.3 | | 5.8 | | 6.6 | 7.6 | | 8.6 | | 10.0 |
| Obesity and HbA1c <53 mmol/mol | 3.6 | 3.9 | | 4.4 | | 5.0 | 5.8 | | 6.8 | | 8.1 |
| No obesity and HbA1c ≥64 mmol/mol | 20.8 | 20.4 | | 20.0 | | 19.3 | 18.6 | | 17.7 | | 16.6 |
| No obesity and HbA1c ≥53 to <64 mmol/mol | 28.4 | 27.9 | | 27.3 | | 26.6 | 25.6 | | 24.5 | | 23.2 |
| No obesity and HbA1c <53 mmol/mol | 32.5 | 32.2 | | 31.7 | | 31.1 | 30.2 | | 29.2 | | 28.0 |
|  | | | | | | | | | | | |
| **HbA1c (mmol/mol)** | **Δ = −10** | | **Δ = −5** | | **Δ = 0** | | | **Δ = +5** | | **Δ = +10** | |
|  | Not treated, % | | Not treated, % | | Not treated, % | | | Not treated, % | | Not treated, % | |
| Obesity (BMI ≥30 kg/m^2^) and HbA1c ≥64 mmol/mol (≥8.0%) | 4.6 | | 4.8 | | 5.2 | | | 5.6 | | 6.2 | |
| Obesity and HbA1c ≥53 to <64 mmol/mol (≥7.0% to 8.0%) | 6.2 | | 6.4 | | 6.6 | | | 6.6 | | 6.3 | |
| Obesity and HbA1c <53 mmol/mol | 6.0 | | 5.6 | | 5.0 | | | 4.6 | | 4.3 | |
| No obesity and HbA1c ≥64 mmol/mol | 16.7 | | 17.7 | | 19.3 | | | 21.3 | | 23.8 | |
| No obesity and HbA1c ≥53 to <64 mmol/mol | 24.6 | | 25.6 | | 26.6 | | | 26.9 | | 26.1 | |
| No obesity and HbA1c <53 mmol/mol | 35.7 | | 33.6 | | 31.1 | | | 28.8 | | 27.0 | |

# **Supplementary Figure 1** Annual prevalence of specific GLP-1 receptor agonist by preparation indicated for diabetes or obesity.


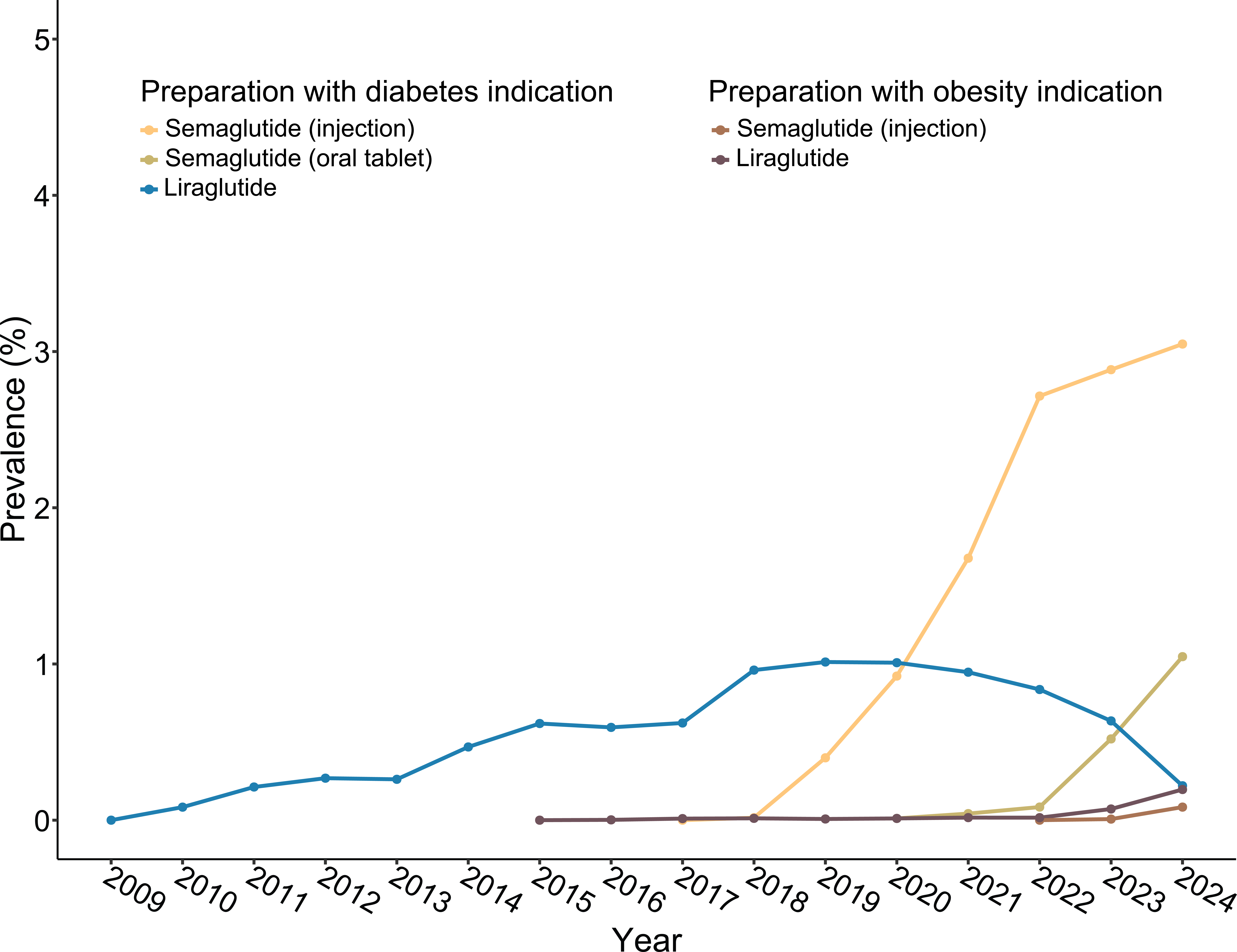


# **Supplementary Figure 2** Cumulative incidence of diabetic ketoacidosis events among new users of SGLT2 inhibitors using an intention-to-treat based exposure.


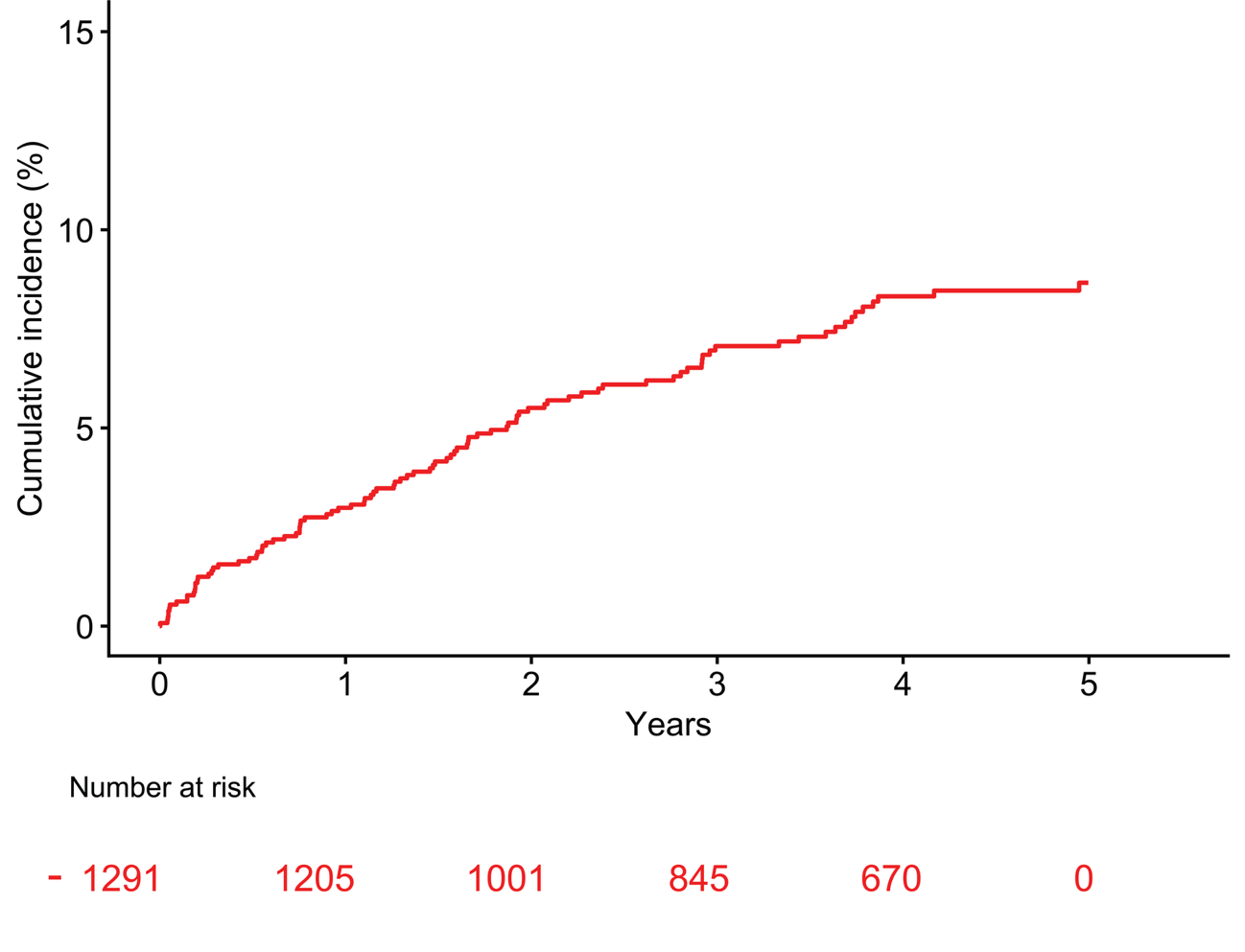

Supplement: Supplementary Figs. S1 and S2 and Tables S1–S8 [file mmc1.docx]
